# Deep Learning Techniques for the Dermoscopic Differential Diagnosis of Benign/Malignant Melanocytic Skin Lesions: From the Past to the Present

**DOI:** 10.3390/bioengineering11080758

**Published:** 2024-07-26

**Authors:** Linda Tognetti, Chiara Miracapillo, Simone Leonardelli, Alessio Luschi, Ernesto Iadanza, Gabriele Cevenini, Pietro Rubegni, Alessandra Cartocci

**Affiliations:** 1 Dermatology Unit, Deparment of Medical, Surgical and Neurosciences, University of Siena, Viale Bracci 16, 53100 Siena, Italypietro.rubegni@unisi.it (P.R.);; 2 Bioengineering and Biomedical Data Science Lab, Department of Medical Biotechnologies, University of Siena, 53100 Siena, Italyernesto.iadanza@unisi.it (E.I.);

**Keywords:** melanocytic skin lesions, melanoma, nevi, atypical nevi, artificial intelligence, deep learning, convolutional neural networks, algorithms, diagnostic models

## Abstract

There has been growing scientific interest in the research field of deep learning techniques applied to skin cancer diagnosis in the last decade. Though encouraging data have been globally reported, several discrepancies have been observed in terms of study methodology, result presentations and validation in clinical settings. The present review aimed to screen the scientific literature on the application of DL techniques to dermoscopic melanoma/nevi differential diagnosis and extrapolate those original studies adequately by reporting on a DL model, comparing them among clinicians and/or another DL architecture. The second aim was to examine those studies together according to a standard set of statistical measures, and the third was to provide dermatologists with a comprehensive explanation and definition of the most used artificial intelligence (AI) terms to better/further understand the scientific literature on this topic and, in parallel, to be updated on the newest applications in the medical dermatologic field, along with a historical perspective. After screening nearly 2000 records, a subset of 54 was selected. Comparing the 20 studies reporting on convolutional neural network (CNN)/deep convolutional neural network (DCNN) models, we have a scenario of highly performant DL algorithms, especially in terms of low false positive results, with average values of accuracy (83.99%), sensitivity (77.74%), and specificity (80.61%). Looking at the comparison with diagnoses by clinicians (13 studies), the main difference relies on the specificity values, with a +15.63% increase for the CNN/DCNN models (average specificity of 84.87%) compared to humans (average specificity of 64.24%) with a 14,85% gap in average accuracy; the sensitivity values were comparable (79.77% for DL and 79.78% for humans). To obtain higher diagnostic accuracy and feasibility in clinical practice, rather than in experimental retrospective settings, future DL models should be based on a large dataset integrating dermoscopic images with relevant clinical and anamnestic data that is prospectively tested and adequately compared with physicians.

## 1. Introduction

### 1.1. Historical Background

The first publication on artificial neural networks (ANNs) appeared in 1943, “A logical calculus of the ideas inherent in neural activity”. The first artificial intelligence (AI) model dates back to 1950, with Alan Turing’s publication “Computing Machinery and Intelligence”, describing how to create intelligent machines; at the time, he had already constructed the well-known AI machine capable of breaking the Enigma code, called “The Bomb”. However, the term “Artificial Intelligence” was officially coined in 1956 during a meeting aimed to create, in two months, a machine capable of simulating every aspect of human learning and intelligence [1].

The first ANN architecture, called Perceptron, was proposed in 1958 by Frank Rosenblatt, the forerunner of today’s ANNs [1,2]. The definition of “Machine Learning” (ML) dates back to the same year, meaning the process that “gives computers the ability to learn without being explicitly programmed” [3]. ML thus involves the creation of algorithms that process data to produce models, which can then recognize patterns, make decisions, or predict outcomes based on new information. The applications of ML are vast and varied, ranging from Natural Language Processing (NLP), where ML helps in understanding and generating human language, to computer vision applications, enabling the ability to interpret visual data from the world, leading to advancements like facial recognition and object detection. Predictive analytics use historical data to forecast future trends, benefiting fields such as stock market prediction and weather forecasting.

Three main elements ushered in the “golden age” of ML: first, the generation of very large amounts of data, “big data”, fostering the search for new computational approaches; second, the development of multiple hardware and software items for analyzing big data and, in parallel, the progressive decrease in their cost; eventually, third, the birth of “Deep Learning” (DL), which was a definition proposed in 1986 to define the subset of ML that incorporates computational models and algorithms that imitate the architecture of human brain networks of neurons (NNs). These models have transformed various fields by enabling computers to detect patterns, make decisions, and predict outcomes with high accuracy [4,5,6].

Briefly, the DL era has seen the birth of convolutional neural networks (CNNs) specialized for processing grid-like data structures, becoming the standard for image-related tasks. Recurrent Neural Networks (RNNs) are specially designed for sequential data; autoencoders (AEs) are used for unsupervised learning in tasks such as dimensionality reduction and anomaly detection. Transformers are designed to handle sequential data, particularly in NLP by using a mechanism called self-attention to weigh the significance of different words in a sentence regardless of their position. Finally, Generative Adversarial Networks (GANs) are based on two competing neural networks, a generator and a discriminator, which are trained simultaneously through adversarial processes for generating realistic images, videos, and even music [2,3,4,5,6,7,8,9,10,11].

In 1998, for the first time, a CNN developed by Le Cun et al. was used to detect handwritten digits and also demonstrated its utility in object and document recognition, while in 2015, their model outperformed human participants in an object classification competition, with an error of 3.6% [7]. CNNs soon evolved into “deep” CNNs (DCNNs) and absorbed image segmentation techniques, creating more complex architectures able to achieve a higher abstraction level and accuracy in feature extraction through image processing [8,9].

CNN/DCNN-based image recognition rapidly became of interest to the industry (employed in automatic car driving for detecting emergency situations using surveillance cameras) [1,2].

Naturally, DCNNs rapidly became of interest as decision support systems for medical image analysis, starting with the neurological and radiological field [10,11,12], and particularly in 2017, when the DCNN ImageNet achieved an error rate of <5% in a Large-Scale Visual Recognition Challenge (ILSVR) competition [13].

Since dermatology is a discipline that fully relies on image recognition, interpretation, and classification to reach a diagnosis, DL models (particularly DCNNs) soon became of interest as decision support systems for dermatologists.

### 1.2. AI Application in Skin Cancer Diagnosis

Malignant Melanoma (MM) is the most aggressive type of skin cancer, representing a significant burden on public health [14]. The data from the International Agency for Research on Cancer (IARC) report a worldwide incidence of more than 330,000 new cases/2022, causing about 58,000 deaths [15].

Starting from 2000, the advent of dermoscopy—either with portable dermatoscopes or fixed videodermatoscopes—has represented a milestone in the early diagnosis of melanoma (MM) and differential diagnosis using clinical simulators. However, dermoscopy accuracy is completely operator-dependent, as it largely varies according to the dermatoscopists’ personal skills. It also requires long-term personal training, and the ability to recognize atypical forms is the prerogative of secondary skin cancer centers dealing with many case studies [16,17].

To give a more standardized approach to this diagnosis, ML models such as “Digital dermoscopy analysis” started to be tested in the early 2000s in experimental settings as decision support systems not only using clinical images, but mostly dermoscopic images, which are standardized in terms of illumination and dimension, presenting the real structure of melanocytic skin lesions (MM, nevi, and atypical nevi) [18,19]. Since 2017, CNN/DCNN models have competed in international challenges on large datasets of clinical or dermoscopic images to reach the best classification power possible [20,21]. Some models have been developed to analyze and classify clinical MM images [22,23,24,25,26,27,28], but the majority of the experiments to date have been dedicated to models trained on dermoscopic images ± clinical images recognition; the main objective was to differentiate MM from benign pigmented cases [29,30,31,32,33,34,35,36].

### 1.3. Current Scenario

The amount of scientific literature in the AI field has dramatically increased ever since, with thousands of records appearing on scientific search engines. Taking a view of the most commonly used search engines, we can observe that the keywords “AI”, “DL”, “ML”, “ANN”, “CNN”, and “DCNN” appear to be often used with overlapping significance; different techniques are often merged together in one paper, although not claimed in the abstract, while in many cases, the work reports on a lesion segmentation/border detection technique and not the diagnostic outcome of the ANN model itself. Moreover, it is not often clear what the database used is (authors’ database or public databases such as those in the ISIC challenge) or what it is composed of. Especially benign cases are often referred to as “no skin cancer” or “benign cases”, but the specific benign diagnoses considered are not reported. Finally, different studies and authors report the experiments using different strategies and describe the results according to different parameters, leading to objective difficulty in comparing the DL models’ performance for a dermatologist reader. When approaching this massive group of merged data, the majority of the review papers produced to date summarize many different AI techniques applied to multiple diagnostic fields, especially skin cancer in general, or report on the results of online international challenges of different computational models [37,38,39,40,41,42].

### 1.4. Aims

On this basis, the present narrative review aimed to screen the scientific literature produced to date on the application of DL techniques to dermoscopic MM/nevi differential diagnosis in order to extrapolate, for the first time, a limited pool of original studies adequately reporting the diagnostic performance of a DL model and comparing them with the clinicians’ performance and/or that of another diagnostic method. The second aim was to compare the selected studies according to a defined set of statistical measures. The third aim was to provide a dermatologist with a comprehensive explanation and definition of the most used AI terms in order to better/further understand the scientific literature of this topic and, in parallel, to be updated on the newest applications in the medical dermatologic field, along with a historical perspective.

## 2. Methods

A thorough literature review was performed in line with the recent recommendations in absence of existing guidelines for narrative reviews [43]. The findings were reported in accordance with the PRISMA (Preferred Reporting Items for Systematic reviews and Meta-Analyses) extension for Scoping Reviews (PRISMA-ScR) Checklist [44].

### 2.1. Information Source

Two search phases were carried out. As a preliminary phase, the Google Scholar search engine was launched to broadly explore all records, including those with only an English written abstract in the fields. In the search phase, we contemporarily used 5 search engines, including those more focused on medical publications (Pubmed, Scopus, and MedRxiv) and those more focused on mathematics, statistics, and engineering publications (ArXiv and WoS). To include all relevant studies, a reference list was checked for any possible article that was ignored by the initial search. The results of the second search phase were compared with those of the first search phase; high-quality papers were selected during each step according to 8 authors’ judgements (see below) and to their appearance in multiple search engines, ensuring high rates of removal. Then, filtering and eligibility phases were performed on this pool of records.

### 2.2. Search

The literature search was carried out for all the articles dealing with DL algorithms that applied to the diagnosis of MM up to 21 May 2024. Three authors (A.C., S.L., and C.M.) were involved in the searching phase and first screening phase. Three authors (L.T., E.I., and A.L.) were involved in the second screening phase. Three authors (G.C, P.R., and L.T.) were involved in the eligibility phase. The titles and abstracts were examined in the search and screening phases, while the whole texts were analyzed in the eligibility phase. In each phase, any eventual disagreement concerning the selection of a record was resolved upon discussion and, if necessary, by consulting an author involved in a different phase. Detailed analysis and data extraction from the final pool of records selected at the end of the eligibility phase was performed by two authors (L.T. and A.C.); the average sensitivity (SE), specificity (SP), accuracy (ACC), and AUC (area under the ROC curve) were calculated.

### 2.3. Eligibility and Exclusion Criteria

**Figure 1:**
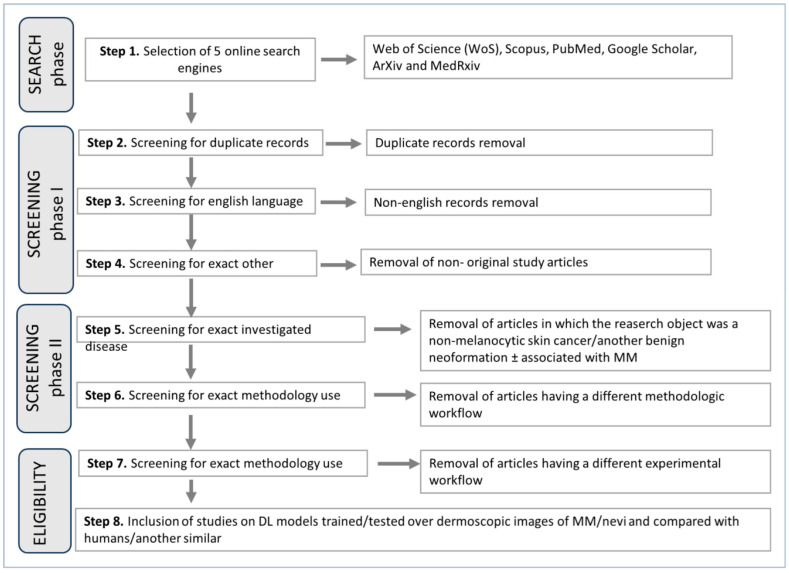
Flow diagram illustrating the search and selection strategies followed for each step.

The search strategy is detailed in Figure 1. The search terms used for each search engine are reported in Table 1. A restriction for English language abstracts, manuscript categories (original papers), topics (MM vs. nevus dermoscopic diagnosis), and methodologies (presentation of one CNN/DCNN trained and tested only on MM/nevi having a predictive outcome) was then applied. Filters in each phase/step were applied by the authors as previously described.

## 3. Results

**Figure 2:**
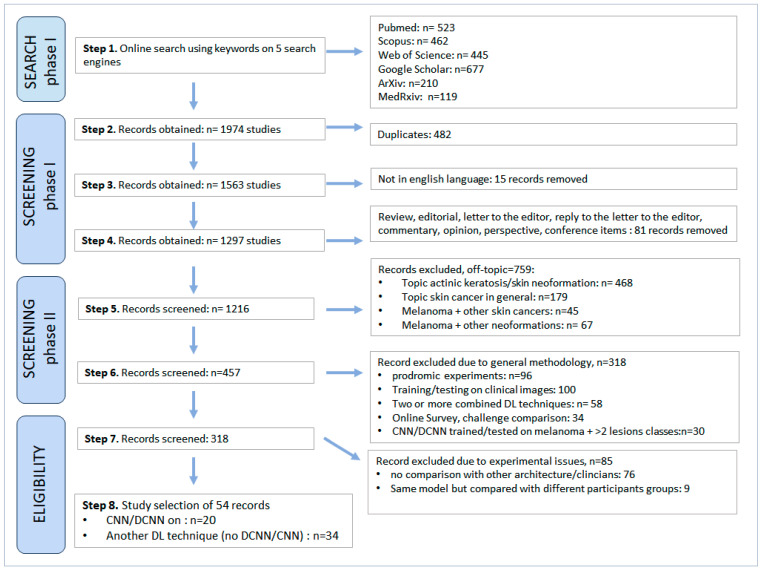
Evidence synthesis. Results of the selection workflow are illustrated as numbers of included records step by step.

The results of the search strategy are synthesized in Figure 2, which also displays the 9 consecutive steps of searching and filtering. At the end of the two screening phases and one eligibility phase, a total of 54 original papers were obtained from the initial 1974 records, i.e., 34 illustrated a DL non-convolutional model, and 20 illustrated a CNN/DCNN. The various findings are discussed and compared below in detail. The computational characteristics of each ML and DL techniques and their definitions are also explained below.

### 3.1. AI Definitions

**Figure 3:**
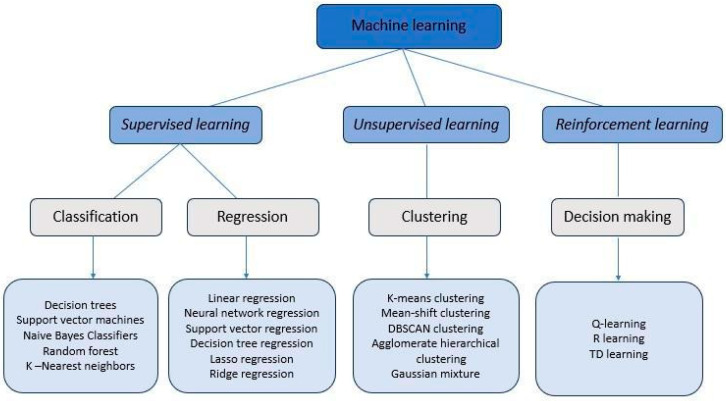
Synthetic scheme summarizing the different machine learning techniques, including deep learning algorithms. (Adapted from ref. [51]).

***Machine learning.*** ML is a subfield of AI that focuses on developing algorithms and statistical models which enable algorithms to learn from data and perform tasks without explicit instructions. The applications of ML are vast and varied, ranging from Natural Language Processing (where ML helps understand and generate human language) to computer vision (which allows systems to interpret visual data from the world, leading to facial recognition and object detection). ML techniques include: (i) *supervised learning*—in the presence of an outcome to be predicted; (ii) *unsupervised learning*—if the aim is to find particular patterns in data; (iii) *semisupervised learning*—used in case of large amounts of data that must be labeled and composed in three steps (a small subset of data is manually labeled, and then a model that learns how to label is developed, and, in the end, this model is used to label the rest of the data); (iv) *reinforcement learning*—algorithms using “trial and error” methods to find optimal strategies, where an agent learns to make consequential decisions by interacting with the environment (i.e., the agent receives rewards or penalties based on its actions, aiming to maximize a cumulative reward over time) [4,5,45,46,47,48,49,50,51] (Figure 3).

***Supervised learning.*** The most common form of ML requires the supervision of human beings feeding the machine with a large set of information, labelling each category, and training the algorithm to recognize these categories. Supervised learning aims to predict an outcome with as little error as possible. Among its applications, support vector machines (SVM)s were successfully used in MM image classification in 2016 in the International Skin Imaging Collaboration ISBI Challenge [20].

***Unsupervised learning.*** In *unsupervised learning*, the machine learns simple concepts, from which it builds abstract concepts. The principal methodologies are “cluster analysis” and “dimensionality reduction”. Cluster analysis is focused on the investigation of subgroups that present similar characteristics based on a multivariate profile. All the cluster techniques are sensible to the number of desired clusters and the chosen type of distance. As an example, the XG boost technique was demonstrated to outperform clinicians in skin cancer detection [52]. Dimensionality reduction techniques are useful in the presence of many variables/observations, especially when expressed in multiple units, to decrease the number of variables by combining them into new ones. It is interesting to recall that human learning is largely unsupervised; we discover the structure of the world by observing it, not by being told the name of every object [47,48,49,50,51].

***Semisupervised learning.*** *Semisupervised learning* is a ML technique that uses a small amount of labeled data and a big amount of unlabeled data during training. This method is effective when labeling data is expensive or time-consuming, yet unlabeled data are abundant. The main idea is to utilize the labeled data to create a model that can make predictions, and then use these predictions to label the unlabeled data iteratively, thus improving the model’s performance. *Semisupervised learning* methods frequently use self-training techniques, in which the model is trained on labeled data before being used to predict labels for unlabeled data. These predictions are then added to the training set. Another prevalent method is co-training, which involves training multiple models on various perspectives of the data and allowing them to teach one another. *Semisupervised learning*, which makes use of both labeled and unlabeled data, can outperform the completely unsupervised learning approaches [5,51,52,53,54].

***Reinforcement learning.*** This refers to algorithms using “trial and error” methods to find optimal strategies, where an agent learns to make consequential decisions by interacting with the environment (i.e., the agent receives rewards or penalties based on its actions, aiming to maximize a cumulative reward over time) [5,51,52,53].

***Deep learning.*** DL maintains the same structure as ML, comprising both supervised and unsupervised techniques, and the principal technique is the artificial NN (ANN). The ANN is a distributed network of computing elements, modeled on a biological neural system and implemented as software. It is capable of identifying the relations in input data that are not easily apparent with the current common analytic techniques. Functioning ANN knowledge is built on learning and experience from the previous input data. On the basis of this priorly acquired knowledge, ANNs can predict relations found in newly presented datasets. ANN models are variegated and currently include CNNs, DCNNs, RNNs, and GANs [49,50,51,52,53,54,55].

**Figure 4:**
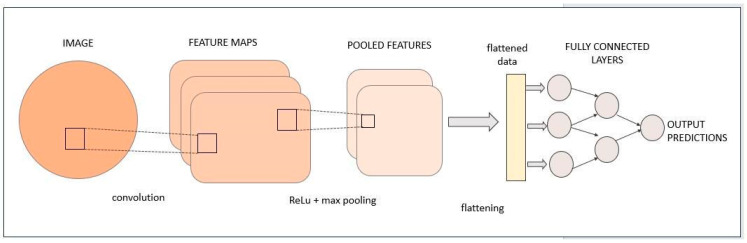
Schematic overview of a CNN/DCNN structure. (Adapted from ref. [51]).

***CNNs.*** CNNs use convolutional layers, along with trainable filters and pooling operations, on raw input images to learn and extract sets of complex high-level/meaningful features automatically. It is possible to create a CNN combining the following layers/functions: convolutional layers (small, learnable filters that slide or “convolve” across the input image to detect patterns like edges, textures, or shapes); pooling layers (used to reduce the spatial dimensions of the feature maps, while retaining essential information); fully connected layers (after feature extraction, these layers connect every neuron to every other neuron in the preceding and subsequent layers, enabling high-level feature combination; one or more fully connected layers are called dense layers); activation functions (applied after each convolutional and pooling layer to introduce non-linearity into the model); a dropout function (regularization technique to prevent overfitting that randomly drops a fraction of neurons during training, reducing the model’s reliance on specific features); and a Loss Function (employed to measure the difference between the predicted and actual values during training). Finally, the output layer produces predictions based on the task at hand; for image classification, it typically has as many neurons as there are classes, so softmax activation is used to convert a raw output into a 0–1 class score. Figure 4 illustrates a common architecture of a CNN. The main three tasks performed by a CNN are image classification (i.e., recognizing what is represented inside the image), image segmentation (i.e., automatically drawing a border around the object represented inside the image), and object detection (i.e., finding specific objects inside the image or video) [45,46,47,48,49,50,51].

***DCNNs.*** DCNNs are CNNs characterized by a very high number of hidden layers, which give them a high level of abstraction and computing power. In parallel, DCNNs need a very large amount of data to be adequately pre-trained before launching the experiment on the dataset of interest. Both CNNs and DCNNs are able to “learn” their own filters in a hierarchical manner that is fully independent of human knowledge [45,46,47,48,49,50]. To date, researchers have employed different available DCNN/CNN architectures, often pre-trained, which were then customized according to the study’s peculiarities. The most commonly used CNN-based architectures include, ordered by the date of launch, the following: Alexnet (2012), GoogleNet Inception v3 [23], Microsoft ResNet-152 [24], GoogleNet Inception v4 [27,28,29,30], Microsoft ResNet-50 [31,32,33], GoogLeNet DCNN [34], VGG, ResNet, DenseNet, and EfficientNet [50]. Each of these models comes with multiple versions. All these architectures aggregate convolutional layers, pooling layers, dense layers, and drop out layers in different ways, while also using different kinds of small, learnable convolutional filters (named “kernels”) [45,46,47,48,49,50,51].

***RNNs.*** RNNs are designed for sequential data, such as time series and natural language; they are a type of artificial neural network designed to analyze a sequential input, where the order of the data points is critical. Unlike standard neural networks, RNNs allow information to persist over time. This architecture makes them very useful for language modelling, speech recognition, and time series prediction. RNNs operate by maintaining a hidden state that stores information about the past inputs. At each time step, they take an input and update the concealed state, thereby “remembering” previous data. This allows them to manage sequences of varying durations and identify trends over time [50,51].

***GANs.*** Generative Adversarial Networks are deep learning frameworks that produce realistic synthetic data. GANs, proposed for the first time in 2014 [53], are made up of two neural networks, the generator and the discriminator, which compete in a zero-sum game. The generator’s role is to generate fictitious data that resemble the actual data distribution. It starts with random noise and converts it to reasonable data samples. The discriminator, on the other hand, assesses these samples and attempts to differentiate between the actual and created data. During training, the generator improves its ability to generate realistic data, and the discriminator improves its ability to detect fakes. Until the generator provides data that are identical to the genuine data, tricking the discriminator, this adversarial process is repeated. There are different types of GAN models depending on the mathematical formulas used and the various ways in which the generator and discriminator interact with each other. Conditional GANs (cGANs) introduce the concept of conditionality, which enables targeted data generation. The generator and the discriminator receive additional information, typically in the form of class labels or other types of conditioning data. For example, if generating images, the condition could be a label that describes the content of the image. The conditioning allows for the generator to produce data that meets specific conditions. Deep Convolutional GANs (DCGANs) integrate CNN architectures into GANs, making them specifically tailored for image processing. With DCGANs, the generator uses transposed convolutions to produce high-level data distributions, and the discriminator also uses convolutional layers to classify the data. The DCGAN also introduces architectural guidelines to make the training more stable. GANs are effective tools for jobs requiring the production of high-quality data since they have been effectively used in a variety of fields, such as image synthesis, video generation, and data augmentation [51,52,53].

### 3.2. Included Studies for Melanoma/Nevi Differential Dermoscopic Diagnosis

A total of 54 studies focused on MM diagnosis, in which the model was trained/tested/validated on dermoscopic images and compared with dermatologists/other similar DL techniques, were finally included. Only 20 studies reported on a CNN/DCNN architecture [29,32,33,54,55,56,57,58,59,60,61,62,63,64,65,66,67,68,69,70], as shown in Table 2. The remaining 34 studies were focused on DL, not the CNN/DNN architecture, and are briefly discussed below [18,19,20,70,71,72,73,74,75,76,77,78].

***DL models (not CNNs/DCNNs).*** These models were developed in early 2000, collectively known as “digital dermoscopy analysis” (DAA) and consisted, generally, of computer-assisted diagnosis (CAD) models, computer vision system (CVM) or support vector machine (SPV) models. In particular, computer-aided detection systems for automatic diagnosis of pigmented skin lesions have been developed by researchers for nearly 30 years. Globally, several studies obtained encouraging results, assuming the computational power available at the time. Generally, the pre-processing phase was given less attention compared with the feature extraction phase. Briefly, the DDA can be considered as the first attempt to move from image color analysis to more complex architectures—of the DL type—combining multiple algorithms, including lesions segmentation, the identification of the region of interest, border detection, and entropy assessment [18,19,20,39,40,41,42,43,79]. Some examples of early models published in 2002 relied on CAD [19,71] software and focused on the nevi/MM differential diagnosis. Piccolo et al. [71] proposed DEM-MIPS software trained on 100 and tested on 341 melanocytic skin lesions (benign/malignant), respectively, able to reach 92% sensitivity (SE) and 74% specificity (SP) compared with the clinical performances of one expert (SE = 92%; SP = 99%) and one resident (SE = 69%; SP = 94%). The DDA model proposed by Rubegni et al. trained on 90 atypical nevi and 57 MMs, evaluating 48 objective parameters, reaching 93% ACC in discriminating the two [19]. Then, in 2011, the updated model (DB-DM-MIPS© System, evaluating 49 objective parameters) proved to be highly performant in a management decision task through a multicentric trial involving 3227 patients across Europe [72] with 91 patients for 10 years.

As per the computer vision system, the one proposed by Friedman et al. in 2008 reached 62% ACC and 98% SE over 99 lesions. Some examples of SVM models for MM/nevi discrimination and management date back to 2010–2015. Tenenhaus et al., 2010 [76], developed a “KL–PLS-based classifier” that when tested on 227, obtained 95% SE and 60% SP compared with their participants’ diagnosis (SE = 70.2%, SP = 83.2%) and therapeutic decision (SE = 86.4%, SP= 56.6%). Ferris et al. [77] tested a DL model on 173 lesions compared with 30 participants (10 dermatologists, 10 residents, and 8 trainees), obtaining 0.81 AUC (SE 96%, SP42.5%). Then, in 2015 [78], Codella et al. elaborated a new approach integrating together DL, sparse coding, and SVM learning algorithms, adopting an unsupervised pattern recognition/feature transfer approach, mimicking the process of expert dermatologists. The proposed model was tested on 334 MM, 144 atypical nevi, and 2146 benign lesions from the ISIC archive, achieving 73.9% accuracy (73.8% SE and 74.3% SP) for the MM/nevi classification task.

***CNN/DCNN models.*** Since 2017/2018, a multitude of experimental models involving CNN/DCNN architecture have been produced in the MM diagnostic field for MM diagnosis. However, according to our filtering strategy, only 20 records turned out to be reporting on original investigations on MM/nevi differential diagnoses performed by CNNs/DCNNs (Figure 2). The main methodological approach is synthesized in Table 2, while Table 3 reports in detailed technical characteristics of each study.

Concerning the computational architecture, a total of eleven records described the CNN architecture [29,32,34,54,55,57,60,62,63,66,69,70], one CNN + ANN architecture [67], and eight DCNNs [56,58,59,61,64,68]. Of note, while fifteen studies report on a different original model, five studies report on the clinical application of the same CNN model, authorized as a medical device, in different subsets of lesions and MM subtypes compared with different groups of clinicians [29,55,63,69,70].

Concerning the pre-training labels, only three studies had the clinical data of the patient integrated with dermoscopic pictures in the training/testing dataset, clearly specified [60,61,67], whereas in two cases, we do not know exactly which kind of clinical data were integrated [29,32]. Of note, only four studies out of twenty had the body sites of the lesions specifically indicated in the dataset [32,61,62,67].

A total of 13 out of 20 studies compared the performances of a proposed CNN/DCNN model with a reader study performed by medical staff (dermatologists/dermatology residents/general practitioners/non-medical personnel/nurses) [29,32,34,54,55,56,59,61,63,68,69,70]. After deriving the ACC values, which were not directly expressed, we estimated that in these studies, the CNN/DCNN models surpassed the humans by +14.85%, showing an average CNN/DCNN-ACC of 87.6% versus an average ACC of 72.75% in the participants’ diagnosis. In 10 out of 13 studies where the SE and SP values were reported, the models obtained an average SE of 79.77% and an average SP of 84.87%. Considering the participants’ SE and SP when reported (12 out of 13 studies), the average SE was 79.78%, and the SP was 69.24%.

Considering the comparison of the proposed CNN/DCNN model with another architecture, we found ten studies. Seven studies compared the CNN/DCNN model only with another architecture (either a CNN or a DCNN) showing an average AUC of 0.902 of the proposed model versus +0.75 AUC, while the participants’ study was not realized [58,60,64,65,66,67]. Three studies compared the CNN/DCNN model with both clinicians’ performances and with another architecture performance on the same tasting dataset, globally showing the overall superiority of the proposed model [56,61,70].

Three studies compared a DCNN [59] or a CNN [32,61] trained with clinical data with the same architecture, but trained with dermoscopic data only, showing an average gain of +5% in accuracy, particularly with +9% in SP. Only one study evaluated the real effect of using AI to correct the intuitive diagnosis of clinicians, with a second-round reading [70], showing increases of +12.3% ACC, +15.8% SE, and +11.6% SP.

Finally, four studies additionally evaluated the management tasks of the participants [29,54,59,61], but only two [59,61] compared them with the model management task. When analyzing these data, it appeared that the participants were poor at making management decisions (excise/follow-up); hence, they sent them the excision-relevant findings on the blinded MM/nevi cases that were much less specific than those of the model for the same lesion (44% SP participants versus 65% SP-DL on average), while the gap in sensitivity was lower (78% SP participants versus 89% SP-DL on average).

Concerning the report of classification performances, some discrepancies were also found. Ten studies had both the AUC and the ACC values indicated, six had only ACC expressed, and sixteen had the SE and SP parameters reported. We derived the ACC values that were possible (18 out of 20 studies), obtaining an average ACC of 83.99%. On average, 16 out of 20 models had the sensitivity and specificity values indicated, resulting in 77.74% SE and 80.61 SP on average.

**Alternative approaches.** Outside the present selection, we found an interesting alternative, recently proposed approach during the search phases [79,80]. Although not fitting the research criteria for the 20 studies of Table 2, it is worth reporting these records for methodological comparison purposes. A paper by Al Sadhan et al. reports on the performance of four unified DCNNs that locate the skin lesions and categorize them into the predefined classes instead of using classification-based solutions. This approach using four DCNN models at the same time (YOLOv3, YOLOv4, YOLOv5, and YOLOv7) was named “You Only Look Once (YOLO) deep learning models”. The experiments carried out over 2750 images from the ISIC dataset (including 374 MM, 1372 N, and 254 seborrheic keratoses) first showed promising results (AUC of 0.91, SE = 86.35%, and SP =85.9%). Another approach is the one that integrated microwave reflectometry and DL imaging for the in vivo diagnosis of skin cancer [80]. The rational riles on the fact that microwave reflectometry can reveal chemical/physical differences between healthy skin and skin with melanoma by interpreting the dielectric properties of biological tissues, known as “dielectric data”. Thus, by integrating microwave reflectometry with CNN-identified features (e.g., asymmetry, irregular borders, abnormal colorations, etc.), the diagnostic accuracy was superior to that of the non-integrated algorithms.

Both the approaches should however be further confirmed in the next future by focusing on the differential diagnosis over melanoma/nevi and by including a human comparison in the clinical setting.

## 4. Discussion

In the last 50 years, the detection and classification of human diseases has been a topic of growing interest for AI research, with a particular focus on oncology [1,2,3,45,46,47,48,49,50]. For example, ML tools (logistic regression and decision trees) and DL tools (DCNNs) have been demonstrated to significantly help physicians in breast cancer detection and monitoring [81,82,83,84]. In dermatology, where the diagnosis largely relies on image interpretation, large attention is paid to skin cancers [81,82,83], and particularly, to MM [38,39,40,85], the most aggressive form. It is characterized by a very good prognosis in the case of early removal. The timely diagnosis of MM relies on the dermoscopic examination in most cases, considering the diffusion of this technique worldwide and its use since 2000 [16,17].

To the best of our knowledge, the present narrative review is the first examining, in detail, only original studies reporting on a DL model applied to the dermoscopic differential diagnosis of MM from nevi/atypical nevi [37,38,39,40,41,42,43,44,45,46,79,80,81,82,83,84,85].

***Limits and weaknesses of DL models tested to date.*** Globally, the DL models proposed since 2017 and tested in experimental settings on skin cancer detection (both on clinical and dermoscopic images) showed a superior or similar performance compared with those of the dermatologists/dermatology residents/general practitioners, taking histology as the gold standard. However, several relevant methodological differences appeared when analyzing these experimental studies; thus, they make any adequate model performance comparison really hard [40]. Moreover, besides the methodological discrepancies, issues in data interpretability, ethical concerns, and different and/or limited clinical validation have been found (Table 2 and Table 3). In particular, by analyzing the 54 studies on DL-based MM diagnosis [18,19,20,29,32,34,54,55,56,57,58,59,60,61,62,63,64,65,66,67,68,69,70,71,72,73,74,75,76,77,78], six main differences were detected, concerning (i) the research team, (ii) the study nature, (iii) the dataset composition, (iv) the computational experiments, (v) the human comparison, and (vi) the comparison with comparison with human participants and/or another model.

- Concerning the composition of the research team, they can be essentially grouped into a non-medical researcher team (e.g., engineers/mathematics/statistics/informatics) and a hybrid team (expert dermatologists collaborating with biomedical engineers/informatic engineers). Consequently, these differences are reflected in many aspects, such as the study methodology, the pre-processing phases, and attention to the data labelling the images. For example, the non-medical teams usually employ large publicly available datasets and achieve high computational power, but miss clinical tests with a human participant group, and/or do not pay attention to the details associated with the dataset (e.g., lesion body location) [18,30,32,58,60,62,64,65,66,67,69,70,71,72,73,74,75,76,77,78]. Technically, those works generally move the basis on the CAD analysis, dedicating large parts of the experiments to the border detection, segmentation, and identification of the region of interest, as well as the widespread use of data pre-processing and image augmentation strategies.
- Regarding the study nature, almost all studies are retrospective, having almost all the lesions tested via histology available, and thus the human decision assisted by DL is virtually deduced [17,18,19,20,21,22,23,24,25,26,27,28]. Moreover, dermatologists recruited for image classification and management tasks do not have the real patient in front of them, but only one dermoscopic picture, or, in a few cases, the picture plus some clinical objective data, while the single lesion history is missing in 98% of studies. Thus, the provided performance results should be interpreted bearing in mind that the study scheme fails to reproduce an in vivo setting.
- The dataset used in the pre-training/training/testing/validation phases is largely variable in terms of image acquisition (tool/conditions), dimension, quality, case selection, and labelling degree. From a technical point of view, dermoscopic and clinical images may differ in size/quality, possible artefacts (pencil marks, rulers/objects, etc.), the device of acquisition, light calibration, etc., and we are not able to understand which patterns the DCNNs/CNNs learn and take into account for the final “decision”, as the process is largely unsupervised. It should be also stressed that some authors use their own datasets for pre-training and testing, some others exploit only one publicly available dataset, while some others use a combination of different public datasets, always choosing a different ratio of MM/nevi/atypical nevi, without any specific explanation in most cases. Furthermore, in some studies, the number of cases does not match the number of lesions/patients not only in the pre-training phase, but also in the training phase; thus, multiple pictures of the same lesion appear to be included in the testing process, altering the final output [59,73]. Concerning clinical dataset characteristics, such as a patient’s phototype, ethnicity, and the body site of the lesion, are almost always not specified, especially in research studies carried out by engineers (without the collaboration of dermatologists). Finally, more and more investigations should be carried out on MM in acral sites, mucosae, or on nails in the future, given that, to date, the used datasets were generally indicated as “body lesions” when indicated.
- Nevertheless, more variability exists in the procedure scheme adopted by different research groups, ranging from pre-processing adopted techniques, segmentation, and feature extraction procedures, and mostly, the construction of the DL architecture (Table 2). The possible combinations in this phase are almost infinite, and we should say, they will persist as an intrinsic feature of this research topic. At present, we can just speculate that one scheme may be more suitable for multiclass classification rather than binary output, but specific comparative work should be carried out in this sense.
- Concerning the comparison with humans, many authors do not plan a “reader study” performed by dermatologists/residents and, when present, all studies report different compositions of these groups in terms of numerosity, professional degree, and, most importantly, dermoscopic skill. Indeed, the experience level should be regarded as the most important parameter influencing a participant’s performance (Table 3).
- Finally, some authors choose to compare the proposed model with the pre-existing ones, and some others do not. If present, the decision on which different architecture to use as a comparison in each original study seems to be totally arbitrary and often driven in order to show the superiority of the proposed model [37,38,39,40,56,58,64,65,66,67].

For these reasons, any generalization derived from meta-analysis/a systematic review should be interpreted with caution [38,39,40].

***Strengths and advantages of CNN/DCNN models tested to date.*** Concerning the small set of 20 studies produced since 2018 and specifically selected according to the research topic (MM/nevi dermoscopic differential diagnosis) and similarity in general methodology (CNN/DCNN), we can make three premises (Table 2 and Table 3).

Firstly, discrete homogeneity can only be found in the pre-training phase, concerning the use of images from the ISIC archive. We can thus speculate that there is surely an under-representation of some ethnic groups in these studies and that those algorithms can be applied only to a certain group of patients/lesions.

Secondly, only two studies specify that the testing and validation dataset included atypical nevi beside MM [57,61], after a pre-training phase with non-atypical and atypical nevi. This leads to the consideration that all the other 18 studies include easy-to-diagnose benign lesions; thus, the CNN/DCNN model accuracy should be interpreted accordingly. Thirdly, only one study can be regarded as a hybrid retrospective prospective from a methodological point of view, showing the effective impact of the CNN’s suggestion on clinicians’ decisions [70].

Taking into account all these premises, looking at the statistical measures derived from the thorough analysis of 17 out of 20 studies, we have a scenario of highly performant DL algorithms, especially in terms of low false positive results, with average values of ACC (83.99%), SE (77.74%), and SP (80:61%) (Table 3).

- Then, in order to speculate if the CNNs/DCNNs were really helpful in a clinical setting, we looked, in detail, at the subset of 13 studies that tested the physicians’ diagnostic abilities to examine the same lesions [29,32,34,54,55,56,59,61,63,68,69,70]. Again, the main difference between algorithms and humans relies on the specificity values, with an +15,63% increase for the CNN/DCNN models (average SP = 84.87%) compared to that of the humans (average SP = 64.24%). Notably, the average sensitivity values of the two groups were very similar, with an SE of 79.77% for the DL models and 79.78% for the humans. According to the reported global performance values, the gap was 14.85% (mean ACC = 87.,6% CNN/DCNN vs. 72.75% of participants).
- As expected, when the participants had the possibility to reformulate their diagnosis based on the DL tool suggestion, they increased not only in SP (+11.6%), but also in SE (+15.8%) [70]; however, other studies are needed to be carried out with this perspective view to clearly demonstrate the usefulness of this kind of algorithm in clinical practice [37,38,39,40,80,81,82,83,84].
- Interestingly, the more relevant clinical patient/lesion data we give to the algorithm to learn, the more specific it becomes (+9% in SP in three studies [32,60,61], with minimal clinical data). Further experiments on larger datasets focused on this specific aim are needed to confirm this hypothesis in the future.

***Future perspectives.*** The use of dermoscopic clinical data for CNN/DCNN training is really a crucial point for this kind of experiment; in general, there is an objective difficulty in reaching a compromise between the data quality (i.e., a thoroughly detailed dataset of cases matching the dermoscopic pictures with the clinical ones and clinical anamnestic relevant data), and, on the other side, the data number (i.e., to reach adequate accuracy, these models require thousands of image cases and different subsets for each developmental phase—pre-training/training/testing/validation). Indeed, only a few specialized centers worldwide are able to set up this kind of integrated/complete dataset and submit them to adequate training and testing, considering that both the dataset collection phase and algorithm creation phases require a long time [17,18,19,20,21,22,23,24,25,26,27,28,29,30,31,32,33,34,35,36,37,38,39,40,61]. Moreover, as this field is a borderland between medicine and mathematics, the aim is to finally apply it to patients and help saving people’s lives with early MM diagnosis. More and more studies generated from the close and continuous collaboration of dermatologists with bioengineers and informatics are needed [37,40,60,61,81].

Finally, the homogenization in study methods and strategies deserves to have comparable studies in the future, paying particular attention to the use of a uniform standard of metrics language and to validation in real-life clinical settings. As shown in this review, no studies were completely uniform in this regard, where the authors chose to use metrics (AUC, accuracy, precision, specificity, recall, false positives/negatives, true negatives/positives, false negatives, positive/negative predictive values, DOR, etc.) essentially according to their preference/technical statistical needs (Table 2). In this sense, position statements and/or recommendations produced by international study groups/task forces variously composed by physicians/dermatologists and bioengineers/informatics/statisticians may be helpful [86].

It is worth noting that, currently, patients seem to rely on diagnostic algorithms more than expert dermatologists do, especially those who are highly skilled in dermoscopic diagnosis, given the diffuse use of smartphone apps/online software for auto-diagnosis/screening/follow-ups [86,87]. In order to make algorithms more familiar to the majority of dermoscopists, preliminary work on the improvement in model specificity should be done. Three parallel strategies may be helpful in this sense. First, we should adopt training and testing methods that simulate, as much as possible, the in vivo setting conditions of a dermatologic. As an example, the possibility to “feed” the model with patients’ macro clinical images/total body photographs/tridimensional images, with a series of relevant anamnesis data and laboratory parameters, and, if understood, with standardized sequential lesional image/data acquired over time should be introduced. Second, involving more and more human intelligence in the second step of the learning phase, reaching a kind of compromise in semisupervised learning, where the model is continuously corrected in those situations and where only humans’ deduction skills succeed, may not only enhance the diagnostic power, but particularly, the management skills of the DL model [59,60]. In this sense, it has been demonstrated that “hive dermatologists” (i.e., multiple experts working together) are more accurate than individual dermatologists and significantly more accurate than a largely validated CNN medical device when tested on images of rare conditions for which the model was not frequently/specifically trained [88]. Third, it would be desirable to perform a long, final validation phase of the model that is carried out exclusively pre-peptically in a *real-life* setting, which is an office of an expert dermatologist.

In the future, hybrid models trained with collective human knowledge derived by the best-performing dermatoscopists may create the generation of hybrid and extremely powerful diagnostic tools. In parallel, future research should clearly investigate and report how the dataset characteristics can influence the model performance and generalizability power [61]. In this sense, the creation of an international online registry integrated with clinical data and the possibility to perform tests in a tele-dermoscopic way may be the response to this problem [37,61].

## 5. Conclusions

On these premises, despite the lack of clinical studies clearly confirming their benefit through investigations on large datasets, including successive clinical decision-making steps, we can be confident in hypothesizing that research advances will make DCNN/CNN tools more and more useful/reliable in the dermoscopic diagnosis of MM using a complex simulator, at least in the near future. Based on the experiments carried out to date, the expected benefits of this future scenario could include a reduction in unnecessary excision due to these tools’ higher specificity compared with that of any dermatologist, with the consequent saving of healthcare resources and money; an increase in the early diagnosis of MM, especially by less-experienced/novice dermoscopists; and a reduction in waiting lists thanks to the possibility of receiving a second opinion in real time, decreasing the number of second confirmatory visits.

## Figures and Tables

**Table 1:** Overview of the key terms used for the search engines during definitive and preliminary search phases.

**Definite search**	**Wos**
	ti = (“Deep Learning” OR convolutional OR dcnn OR cnn OR cnns OR dcnns OR rcnn) AND ti = (“skin lesion*” OR “skin defect*” OR nevus OR nevi OR melanocytic OR “skin cancer” OR melanoma OR “skin tumor*” OR “skin tumour*” OR “skin neoplasm*” OR “cutaneous cancer” OR “cutaneous tumor*” OR “cutaneous tumour*” OR “cutaneous neoplasm*” OR dermoscopy OR dermoscopic OR dermatoscopy OR dermatoscopic).
	**Pubmed**
	(“Nevi and Melanomas”[Mesh]) AND (“Deep Learning”[Mesh]) OR (“Deep Learning”[ti]) OR convolutional[ti] OR dcnn[ti] OR cnn[ti] OR cnns[ti] OR dcnns[ti] OR rcnn[ti] AND (“skin lesion*”[ti] OR “skin defect*”[ti] OR nevus[ti] OR nevi[ti] OR melanocytic[ti] OR “skin cancer”[ti] OR melanoma[ti] OR “skin tumor*”[ti] OR “skin tumour*”[ti] OR “skin neoplasm*”[ti] OR “cutaneous cancer”[ti] OR “cutaneous tumor*”[ti] OR “cutaneous tumour*”[ti] OR “cutaneous neoplasm*”[ti] OR dermoscopy[ti] OR dermoscopic[ti] OR dermatoscopy[ti] OR dermatoscopic[ti]).
	**ArXiv, MedRxiv**
	“deep convolutional/convolutional neural network and melanoma/skin cancer/skin lesions/melanocytic lesions”, “deep learning and dermatology/dermoscopy”, “automated classification/detection and dermatology/dermoscopy”, “image classification and melanoma/melanocytic lesions/dermoscopy”.
	**Scopus**
	TITLE (“Deep Learning” OR convolutional OR dcnn OR cnn OR cnns OR dcnns OR rcnn) AND TITLE (“skin lesion*” OR “skin defect” OR “squamous cell” OR nevus OR nevi OR melanocytic OR “skin cancer” OR melanoma OR “basal cell carcinoma*” OR “skin tumor*” OR “skin tumour*” OR “skin neoplasm*” OR “cutaneous cancer” OR “cutaneous tumor*” OR “cutaneous tumour*” OR “cutaneous neoplasm*” OR dermoscopy OR dermoscopic OR dermatoscopy OR dermatoscopic)
**Preliminary search**	**Google Scholar**
	(“Deep Learning” [Mesh] OR “deep-learning” OR “deep-learning” OR “deep neural networks” OR ““deep neural network” or ((deep OR machine* OR convolute*) AND (learn* OR neural*)) OR “convolutional neural network” OR CNN* or “Artificial Intelligence* [Mesh] OR “artificial intelligence” OR “artificial-intelligence” OR AI [Title/Abstract] OR “Machine Learning”[Mesh] OR “Neural Networks, Computer” [Mesh] OR melanoma* OR melanoma diagnosis* OR (melanoma*) AND (deep learning*)) OR (convolutional neural network*) AND (melanoma*) AND (nevus*))

**Table 2:** Comparison of methodologies and performances of 20 CNN/DCNN architecture designed for melanoma/nevi differential diagnosis.

Year	Authors	Ref	Dataset Used	Clinical Data + Dermoscopic Images	Diagnostic Testing by Participants	Management Study of the cnn/dcnn	Management Study of Participants	Comparison with Another DL Architecture
*Details*			*training/testing/validation*	*yes/no*	*yes/no*	*yes/no*	*yes/no*	*yes/no*
2018	Haenssle HA, et al.	[54]	*training, testing, validation*	no	yes	no	yes	no
2018	Yu C, et al.	[55]	*training, testing*	no	yes	no	no	no
2019	Chandra TG, et al.	[56]	*training, testing, validation*	no	yes	no	no	yes
2019	Binker T, et al.	[57]	*training, testing, validation*	no	yes	no	no	no
2019	Brinker, T. et al.	[34]	*training, testing, validation*	no	no	no	no	no
2019	Abbas Q, et al.	[58]	*training, testing*	no	no	no	no	yes
2019	Phillips M, et al.	[59]	*training, testing*	no	no	yes	yes	no
2019	Gonzalez-DIaz, et al.	[60]	*training, testing, validation*	yes	yes	no	no	yes
2020	Tognetti L, et al.	[61]	*training, testing, validation*	yes	yes	yes	yes	yes
2020	Lee S, et al.	[32]	*training, testing*	no	yes	no	no	yes
2020	Winkler JK, et al.	[62]	*training, testing*	no	no	no	no	no
2020	Fink C, et al.	[29]	*training, testing*	no	yes	no	yes	no
2020	Han, et al.	[63]	*training, testing*	no	yes	no	no	no
2020	Adegun A., et al.	[64]	*training, testing*	no	no	no	no	yes
2020	Grove R, et al.	[65]	*training, testing*	no	no	no	no	yes
2021	Nasiri S, et al.	[66]	*training, testing, validation*	no	no	no	no	yes
2020	Ningrum DN, et al.	[67]	*training, testing, validation*	yes	no	no	no	yes
2021	Pham, et al.	[68]	*training, testing*	no	yes	no	no	no
2022	Winkler JK, et al.	[69]	*training, testing*	no	yes	no	no	no
2023	Winkler JK, et al.	[70]	*training, testing*	no	yes	yes	no	no

**Table 3:** Comparison of methodology and experimental details of the 20 selected studies on CNN/DCNN architecture designed for melanoma/nevi differential diagnosis.

Ref	Architecture	DL Model	Dermoscopic IMAGE Dataset Pre-Training	Training Dataset	Testing Dataset	Validation Dataset	Model Output	Body Site of Application
	***original/available format***	**CNN, DCNN, RNN**	***Public/institutional/own***		***Public/institutional/own***		***Binary/continuous***	***Details***
[54]	Google’s Inception v4	CNN, pretrained on 1000 images	ISIC archive	300 images (34 MM + 266 N)	100 images	100 images (80 MM + 20 nevi)	continuous 0–1	unspecified
[55]	MatConvNet, modified, VGG model with 16 layers			53	MatConvNet, modified, VGG model with 16 layers	/	binary otuput (N/MM)	palms and soles
[56]	original scheme, 14 layers	DCNN	ISIC archive	1643 images (773 N + 870 MM)	400 images (200 N + 200 MM)	156 N + 44 MM	binary otuput (N/MM)	unspecified
[57]	ResNet50	CNN	ISIC archive + HAM10000 dataset;	4204 images (1888 MM + 2316 AN	1200 images (800 N + 200 MM) ratio MM/N = 1:4	1359 images (230 MM + 1129 AN); ratio MM/N = 1:14	continuous 0–1	unspecified
[34]	ResNet50	CNN	ISIC archive + HAM10,000: 20735: images	12,378 images	100 images	1,259 images (MED-NODE database + clinical images)	binary otuput (AN/MM)	unspecified
[58]	fusion of multiple feature CAD system + DCNN + RNN	DCNN, “DermoDeep”, original	ISIC archive (1600) + (500) + Skin-EDRA dataset + Ph2-dataset (100) + DermNet (600)	2800 images (1400 N + 1440 MM)	2800 images (1400 N + 1440 MM)	/	binary otuput (N/MM)	unspecified
[59]	original scheme	DCNN	1550 images: 551 biopsied (125 MM + 148 AN + 278 other) + 999 controls not biopsied (Public: not specified)	858 images (36 MM, 253 not MM) (istitutional dataset)	731 images (51 MM)	/	continuous 0–1	unspecified
[60]	ResNet50	CNN, “DermaKNet”, original	2017 ISBI Challenge + EDRA dataset + ISIC	2000 images (374 MM, 1372 N, 254 SK) ± age/sex	150 images ± age/sex data	600 images ± age/sex metadata	binary output (MM vs. N; MM vs. SK)	unspecified
[61]	ResNet50	“iDCNN_aMSL”	ISIC archive: 20735 images (18566 N + 2169 MM)	630 images (429 AN + 201 EM) ± age/sex/diameter/anatomy site clinical data (iDScore_body dataset)	214 images (140 AN + 74 EM) ± age/sex/diameter/anatomy site clinical data (iDScore_body dataset)	135 images (93 AN + 42 EM) ± age/sex/diameter/anatomy site clinical data (iDScore_body dataset)	continuous 0–1	Body (no face, palms, soles)
[32]	ResNet 50	CNN “ALM-net”	own: 1072 images of MM and N	872 images N + MM ± clinical data (unspecified)	200 images ± clinical data (unspecified)	/	binary otuput (N/MM)	palms and soles
[62]	Google’s Inception v4	“Moleanalyzer-Pro® CNN”	istitutional (50000 images)	NA	180 MM, 600 nevi (363 biopsied, 210 followed-up, 27 consensus)	6 subsets, each including 100 N + 30 MM)	NA	SSM, LMM, mucosal MM, NM, nailMM, AMM,
[29]	Google’s Inception v4	“Moleanalyzer-Pro® CNN”	istitutional: 129,487 images + labels	115,099 images N + MM	72 images (36 MM + 36 CN)	/	binary otuput (combined N vs. MM)	unspecified
[63]	Microsoft ResNet 152	CNN	224,181 images (public + istitutional)	220, 680, 174 disease classes	/	/	binary otuput (CN/MM)	
[64]	original scheme	DCNN (“Deep Convolutional EncoderDecoder Network”)	ISIC 2017, PH2 datasets		/	/	binary otuput (N/MM)	unspecified
[65]	ResNet 50		ISIC archive + “UDA1, UDA2, MSK-2, MSK-3, MSK-4" databases	3222 images (2361 N + 591 MM) (ImageNet)	77 images (27 MM + 50 N) (“Dermnet NZ”)		binary otuput (N/MM)	unspecified
[66]	original	CNN (“DePicT Melanoma Deep-CLASS”)	ISIC archive, 400 images	1346 images N + MM (ISIC archive)	1796 images N + MM (ISIC archive)	450 images N + MM (ISIC archive)	binary otuput (N/MM)	unspecified
[67]		CNN + ANN	“ISIC, HAM 10000, MSK-1, MSK-2,MSK-3,MSK-4”	900 (281 MM + 619 N) + clinical data (age, sex, anatomic site)	300 images (93 MM + 207 N) + clinical data (age, sex, site)	180 images + clinical data (age, sex, anatomic site)	binary otuput (N/MM)	body + head/neck
[68]	wInceptionV314, ResNet5015, Dense- Net16916	DCNN	ISIC 2019: 17302 images (4503 MM + 12,799 N)	1730 images (450 MM + 1280 N) (MClass-D dataset)	NA	59 high-risk patients	binary otuput (N/MM)	unspecified
[69]	GoogleNet Inception v4	“Moleanalyzer-Pro® CNN”	M10000 dataset + institutional dataset	150000 images	236 images		continuous 0–1	unspecified
[70]	GoogleNet Inception v4	“Moleanalyzer-Pro® CNN”			228 images (190 N + 38 MM)		continuous 0–1	unspecified
**Ref**	**Model Performance**	**Model Management**	**Participants**	**Participants’ Skill Level**	**Participants’ Performance**	**Participants’ Management**	**Comparison with Performances of the Other Models/Checklists Tested on the Same Dataset**	
	***AUC %; SE%; SP%: ACC (%), PPV, NPV, DOR***		***n, Profession***	***Years/Experience in Dermoscopy***	***AUC %; SE%; SP%: ACC (%), PPV, NPV, DOR, PRECISION***			
[54]	AUC = 0.95; SE = 63.8%; SP = 86%	NA	58 dermatologists	17 with <2 years, 11 with 2–5 years, 30 with ≥5 years	only dermoscopy: ACC = 79%; SE = 86.6%, SP = 71.3%. clinic + dermoscopy: ACC = 82%, SE = 88.9%, SP = 75.7%	only dermoscopy: ACC 0.82%; SE 98.8%, SP64.6%. clinic + dermosc: ACC = 0.83%,SE = 9844%6%, SP 66.7%	**/**	
[55]	AUC = 0.835; SE = 92.57%, SP = 75.39%	NA	2 general practicioners, 2 dermatologists	2 beginners, 2 experts	Experts: ACC = 81.08%; Beginners: ACC = 67.84%	/	/	
[56]	AUC = 0.817; SE = 75%; SP = 88%	NA	dermatology residents	2nd and 3rd year of residency	ACC = 87%; SE 85.2%; SP 60.9%	NA	Automatic Multi-Layer Perceptron (MLP): ACC = 76%, SE 70.5%, SP = 87.5%; ABCD rule: AUC = 56.10%, SE = 78.1%, SP = 45.7%	
[57]	NA	NA	145 (142 dermatologist, 3 residents)	100 with >10 years, 15 with 5–10 years, 85 with <5 years	Avg ACC = 76.9%; SE = 67.2%, SP = 62.2%	NA	/	
[34]	SE = 82.3%, SP = 77.9%	NA	157 (52 dermatologists, 92 residents	NA	SE = 74.1%, SP = 60%	NA	/	
[58]	AUC = 0.96; SE = 93%; SP = 95%; ACC = 95%	NA	/	/	/	/	DCNN: “Jeremy_deep”: 82%, 78%, 80%, 79%; “Premaladha_deep”: 84%, 80%, 83%, 82%	
[59]	average AUC = 0.918; SE = 100%/SP = 78.1%	NNB = 6.83 on average	/	/	ACC = 77.8; SE = 95%, SP = 69.9% (over 1582 images)	NNB 4.92, PPV 20.3%, NPV100%	NA	
[60]	AUC 0.873; MM vs. N); 95.2% MM vs. SK	NA	/	/	/	/	“DermaNet” (without clinical data): AUC 85.6%, MM vs. N); 95.6% MM vs. SK	
[61]	AUC = 0.903; SE = 86.5; SP = 73.6%	SE = 89, SP = 73.5%	111 (65 dermatologists,46 residents), (63 F, 48 M) residents.	45 with >8 years, 20 with 5–8 years, 37 with 1–4 years, 9 with <1 years,	ACC = 69.2%, SE = 77%, SP = 61.4%	SE = 78%, SP = 21%	DCNN_aMSL (no clinical data): diagnosis:AUC 86.6%, SE 89.2%, SP 65.7%. Management: SE = 86%, SP = 65.7%	
[32]	AUC = 0.976; SE = 90%; SP = 95%; ACC = 92.5%	NA	60 (20 dermato-logists, 20 residents, 20 general pract)	NA	ACC = 74.7%; SE = 79.9%; SP = 69.5%;	/	model with no clinical data: SE = 88.7%, SP = 85%, ACC = 86.9%	
[62]	SSM/NM: AUC0.98; LMM: AUC 0.926; AMM: AUC 0.928; mucosal MM: AUC 0.75; nail MM: AUC = 0.621	NA	/	/	/	/	/	
[29]	SE = 97.1%, SP = 78.8%; DOR = 34 (95% CI [4.8–239]	NA	11 dermatologists	Beginner: <2 years (3), Skilled:2–5 years (5) Expert: ≥5 years (3)	SE 90.6%; SP = 71%, DOR = 24 (95% CI [11.6–48.4]	SE 100%, SP 47.6%	/	
[63]	SNU AUC 0.937 ± 0.004 Edinburgh AUC 0.928 ± 0.002	NA	70 (21 dermatologist, 26 residents, 23 nonmedical		Dermatologists SE 77.4% ± 10.7 SP 92.9% ± 2.4 AUC 0.66 ± 0.08		/	
[64]	segmentation: ACC = 95%, SE = 95%, SP = 95.5%	NA	/	/	/	/	“U-Net”: ACC = 93%, SE = 82%, SP = 97%ResNet: ACC 93%, SE 80%, SP: 98%	
[65]	ACC 86.7% (SE = 81.4%, SP = 92%)	NA	/	/	/	/	DenseNet169:80% ADDI CNN:97.5%	
[66]	ACC = 75%, SP = 78%	NA	/	/	/	/	“DePic T Melanoma CLASS”: AUC 0.68	
[67]	AUC = 0.971; Precision = 94.33%, recall = 87.1%, ACC 97.10%	NA	/	/	/	/	Same CNN model: AUC = 0.82; precision = 81.67%, RECALL = 52.7%, ACC 81.67%	
[68]	AUC = 0.94, SE = 85%, SP = 95%	NA	157 dermatologists	42 with >12 years, 32 with 4–12 years, 37 with 2–4 years, 46 with <2 years	ACC = 67.1%, SE = 74.1%, SP = 60%	NA	/	
[69]	baseline AUC = 60.69 (SE = 25.4%, SP = 92.7%) Follow-up: AUC = 81.7% (SE = 44.1%, SP = 92.7%)	NA	26 dermatologists	different skill levels	ACC = 40.7%, SE = 66.1% SP = 55.4%	NA	/	
[70]	ACC = 87.7%, SE = 81.6%, SP = 88.9%	ACC = 63%, SE 100%, SP = 55.8%	22 dermatolgogists	78 lesions examined by dermatologists with <2 years, 96 lesions by derm with 2–5 years, 54 lesions by derm with >5 years	ACC = 74.1%, SE = 84.2%, SP = 72.1%	NA	Dermatologists + CNN: AUC = 86.4%, SE 100%, SP = 83.7%	

## Data Availability

Data are available from the corresponding author upon reasonable request.
